# Meta-analysis of global prevalence of hepatitis E virus infection in deer

**DOI:** 10.3389/fmicb.2025.1741279

**Published:** 2025-12-19

**Authors:** Zhen-Qiu Gao, Guang-Rong Bao, Hai-Tao Wang, Yong-Jie Wei, Miao Zhang, Wen-Xu Tan, Hong-Lang Liu, Quan Zhao, Qing-Long Gong, Jing Jiang, Ya Qin

**Affiliations:** 1 School of Pharmacy, Yancheng Teachers University, Yancheng, Jiangsu, China; 2 College of Life Science, Changchun Sci-Tech University, Shuangyang, Jilin, China; 3 College of Veterinary Medicine, Qingdao Agricultural University, Qingdao, Shandong, China; 4 College of Veterinary Medicine, Jilin Agricultural University, Changchun, Jilin, China; 5 Lvliang Municipal Bureau of Agriculture and Rural Affairs, Lvliang, Shanxi, China

**Keywords:** deer, HEV, Meta-analysis, prevalence, risk factors

## Abstract

**Introduction:**

Hepatitis E virus (HEV) is an important zoonotic pathogen, and deer serve as a potential wildlife reservoir capable of contributing to human infections through wildlife–livestock–human transmission pathways. Accurate estimates of HEV prevalence in deer are essential for understanding zoonotic risks and informing surveillance strategies.

**Methods:**

We conducted a systematic search across five major databases. Among 134 publications screened, 33 studies met the inclusion criteria for meta-analysis. Pooled prevalence estimates were calculated, and subgroup analyses were performed by region, development status, time period, age, sex, and diagnostic method. Serological assays (ELISA) and molecular assays (RT-PCR and RT-qPCR) were evaluated separately.

**Results:**

Significant geographical and demographic variation in HEV prevalence was observed. North America showed the highest pooled prevalence (29.57%, 95% CI: 0.00–89.25), with Mexico reporting the highest national estimate (62.68%, 95% CI: 54.54–70.47). Developing countries exhibited higher prevalence (21.45%, 95% CI: 7.12–40.63) than developed countries (5.01%, 95% CI: 2.40–8.43). HEV infection decreased over time, with lower prevalence after 2010 (4.92%, 95% CI: 1.57–9.83) compared with before 2010 (13.17%, 95% CI: 6.01–22.45). Adults had higher infection rates (23.02%, 95% CI: 9.04–41.06) than juveniles (10.11%, 95% CI: 5.83–15.41), and females slightly exceeded males (6.25% vs. 5.07%). Serology indicated past exposure (10.48%, 95% CI: 4.29–18.92), while molecular methods reflected active infection, with pooled rates of 8.58% for RT-PCR and 5.22% for RT-qPCR.

**Discussion:**

Our findings reveal substantial heterogeneity in HEV prevalence among deer and highlight the importance of standardized diagnostic protocols. These results underscore the need for harmonized surveillance to better assess zoonotic risks and support public health strategies targeting HEV transmission at the wildlife–human interface.

## 1 Introduction

Hepatitis E virus (HEV) is a non-enveloped RNA virus in the family *Hepeviridae*, now classified by ICTV (2022/2023) under the genus *Paslahepevirus*, species *Paslahepevirus balayani* (Pavlova et al., 2025). The classification of HEV can be further refined into eight distinct genotypes, namely HEV1 to HEV8 (Raji et al., 2022). HEV1 and HEV2 exclusively infect humans (Kamar et al., 2017). Genotypes HEV3 and HEV4, which can infect both humans and animals, are mainly found in developed areas and pose a risk of zoonotic transmission (Kamar and Pischke, 2019). The primary mode of transmission for HEV is fecal-oral, often through water contaminated with feces. Additionally, consumption of contaminated food such as pork and milk or healthcare-associated means can also lead to transmission (Dziedzinska et al., 2020; Hu et al., 2025). Human infection with HEV typically results in an acute, self-limiting hepatitis. However, in certain individuals—particularly those who are immunocompromised—the infection can progress to chronic hepatitis and may further lead to complications such as cirrhosis or liver failure (Kamar et al., 2017).

In recent years, HEV detection has been reported in a wide range of domestic and wild animals. Several systematic reviews have summarized HEV infection and RNA detection among ruminants, highlighting growing evidence of HEV exposure in cattle, goats, sheep, deer, and other wildlife species (Spahr et al., 2018; Wang et al., 2025). These studies emphasize the potential role of ruminants in HEV ecology but also reveal substantial variation in detection rates across species and regions. Notably, although deer are increasingly recognized as potential reservoirs—supported by reported human infections linked to consumption of raw deer meat (Tei et al., 2003)—no previous review has provided a focused, quantitative assessment of HEV prevalence specifically in deer populations worldwide.

Therefore, this systematic review and meta-analysis aim to evaluate the global prevalence of HEV infection in deer and to explore factors influencing its occurrence, thereby filling an important knowledge gap within the broader context of HEV epidemiology in ruminant hosts.

## 2 Materials and methods

### 2.1 Search strategy

This systematic review and meta-analysis followed the PRISMA 2020 guidelines (Page et al., 2021), and the completed PRISMA 2020 checklist is available as Supplementary Material 1. A comprehensive search was conducted in five databases (CNKI, VIP, Wanfang, PubMed, and ScienceDirect) for studies published in English or Chinese up to October 2025. Search terms combined controlled vocabulary (e.g., MeSH) and free-text keywords related to deer and hepatitis E virus using Boolean operators. For PubMed, the strategy was:

(“Deer”[MeSH] OR deer OR cervid) AND (“Hepatitis E”[MeSH] OR “hepatitis E virus” OR HEV). Equivalent simplified strategies were adapted for other databases as follows:

ScienceDirect: (deer OR cervid) AND (“hepatitis E” OR “hepatitis E virus” OR HEV);

CNKI, Wanfang, VIP: (Deer OR Deer family OR Deer genus) AND (Hepatitis E virus OR HEV).

### 2.2 Literature inclusion and exclusion criteria

Studies were included if they met all of the following criteria: (1) Target population: deer species (e.g., red deer, roe deer, sika deer, fallow deer). (2) Outcome: HEV infection detected by validated laboratory methods (e.g., ELISA, RT-PCR, sequencing). (3) Study design: cross-sectional or surveillance studies providing prevalence data. (4) Language: English or Chinese. (5) Sufficient data to extract sample size and number of positive cases.

Exclusion criteria: (1) Reviews, case reports, conference abstracts, or experimental infection studies; (2) Studies lacking primary prevalence data; (3) Studies on non-cervid species; (4) Duplicate datasets.

### 2.3 Data extraction and quality evaluation

The process of data extraction and recording was carried out independently by two researchers, with any disagreements or uncertainties being resolved by the lead author of the study. The extracted information includes the article title, first author, publication year, species, detection method, regions, feeding practices, gender, age, sampling year, country, total number of tests conducted and number of positive results obtained. These extracted data were recorded and used to establish a comprehensive database.

Methodological quality was assessed using the Joanna Briggs Institute (JBI) Critical Appraisal Checklist for Prevalence Studies, a validated tool widely used in meta-analyses. The checklist includes nine domains evaluating sampling methods, sample size adequacy, measurement validity, statistical analysis, and response rate. Each study was rated as high, moderate, or low quality.

### 2.4 Statistical analysis

The software package “meta” (version 6.5–0) of R software (version 4.3.1) is used to calculate and analyze the collected data. In order to make the analyzed data more close to Gaussian distribution, raw rate (PRAW), log conversion (PLN), logit conversion (PLOGIT), arcsine conversion (PAS) and double arcsine conversion (PFT) were used for data conversion, and the conversion rate was based on Shapiro–Wilk normal test. *W* values close to 1 and *p* values greater than 0.05 are close to the Gaussian distribution criterion.

Heterogeneity was assessed using Cochran’s Q, *I*^2^, and *χ*^2^. We considered heterogeneity to be substantial when *P* (from Cochran’s Q) < 0.05 and *I*^2^ > 50%; in such cases we used a random-effects model. Otherwise, a fixed-effect model was considered. In practice, given the expected variability in prevalence estimates by geography and method, we used a random-effects model for the primary analyses. The forest plot visualizes the statistical results of the meta-analysis.

Publication bias is determined by the symmetry of the funnel plot. Egger’s *p* < 0.05 indicates publication bias. Sensitivity analysis evaluated the stability of the meta-analysis model and the reliability of the results. Subgroup analysis and univariate regression analysis were used to explore the sources of heterogeneity among the included studies and to predict the factors contributing to heterogeneity, as well as the effects of different risk factors on HEV infection in deer.

## 3 Results

### 3.1 Results of literature search and quality assessment

**Figure 1:**
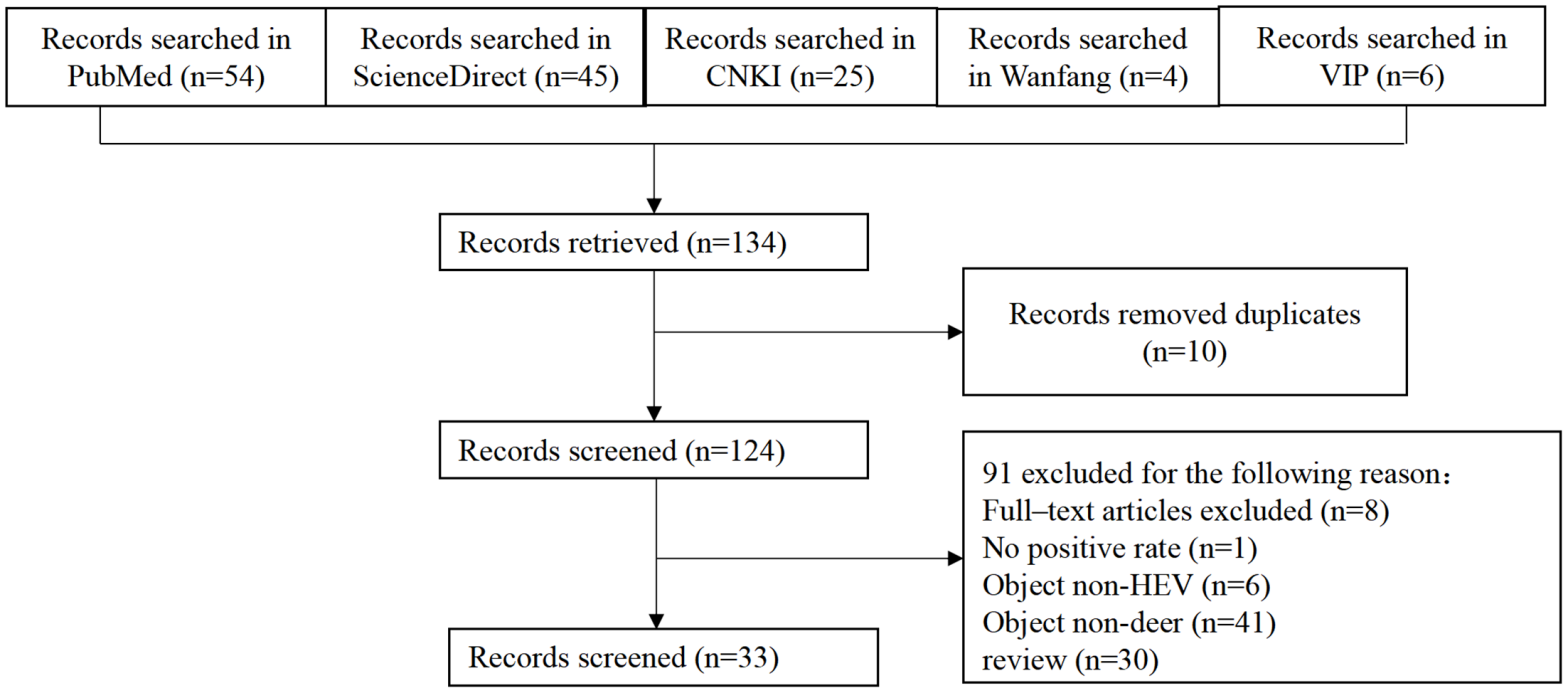
Flow diagram of literature search and selection.

A total of 134 literature were retrieved from 5 literature databases and 33 were finally included after screening (Figure 1, Table 1). Among them, there are 10 articles with a score of 2, 17 articles with a score of 3, 4 articles with a score of 4, and 2 articles with a score of 5 (Table 1).

**Table 1:** Basic information of included studies.

Study ID	Sampling time	Country	Species	No. Positive/No. Tested	Sample type	Detection Method	Genomic regions	Feeding practices	Genotypes
Sonoda et al. (2004)	2003–2004	Japan	Deer (unknown species)	2/117	Serum and liver	ELISA	–	Wild	–
Qu et al. (2006)	UN	China	Deer (unknown species)	348/798	Serum	ELISA	–	Culture	–
Reuter et al. (2009)	2001–2006	Hungary	Roe deer (*Capreolus capreolus*),	11/32	Feces, liver and intestinal	RT-PCR	ORF2	Wild	HEV-3
Tomiyama et al. (2009)	2006–2007	Japan	Deer (unknown species)	181/520	Serum	ELISA	–	Wild	–
Forgach et al. (2010)	2005–2009	Hungary	Roe deer (*Capreolus capreolus*), Red deer (*Cervus elaphus*)_	12/71	Faeces and liver	RT-PCR	ORF1/ORF2	UN	HEV-3 (3e)
Boadella et al. (2010)	2000–2005	Spain	Red deer (*Cervus elaphus*)_	101/968	Serum	ELISA	–	Wild/semi-Wild	–
Rutjes et al. (2010)	2006–2008	Holland	Red deer (*Cervus elaphus*)_, Roe deer (*Capreolus capreolus*)	6/47	Serum,liver and faecal	RT-qPCR	ORF2	Wild	HEV-3
Medrano et al. (2012)	2004–2009	Mexico	White-tailed deer (*Odocoileus virginianus*)	89/142	Serum	ELISA	–	Culture	–
Lhomme et al. (2015)	2010–2011	France	Deer (unknown species)	2/62	Liver and bile	RT-qPCR	ORF3	Wild	UN
Serracca et al. (2015)	2012–2014	Italy	Roe deer (*Capreolus capreolus*),	0/30	Liver	RT-PCR	ORF2	Wild	UN
Zhang et al. (2015)	2012–2013	China	Sika deer (*Cervus nippon*)	46/847	Serum	ELISA	–	UN	–
Kukielka et al. (2016)	2003–2010	Spain	Red deer (*Cervus elaphus*)_	9/70	Serum	ELISA	–	Wild	–
Neumann et al. (2016)	2000–2012	Germany	Red deer (*Cervus elaphus*)_, Roe deer (*Capreolus capreolus*), Fallow deer (*Dama dama*)	14/361	Serum and liver	ELISA	–	Wild	–
Di Bartolo et al. (2017)	2007–2010	Italy	Red deer (*Cervus elaphus*)_	10/91	Serum	RT-PCR	ORF2	Wild	HEV-3 (3e)
Weger et al. (2017)	1990–1991	Canada	White-tailed deer (*Odocoileus virginianus*), Mule deer (*Odocoileus hemionus*), Reindeer (*Rangifer tarandus*)	30/534	Serum	RT-PCR	ORF1	Wild	UN
Anheyer-Behmenburg et al. (2017)	2013–2014	Germany	Roe deer (*Capreolus capreolus*), Red deer (*Cervus elaphus*), Fallow deer (*Dama dama*)	7/183	Serum	RT-qPCR	ORF1	Wild	HEV-3 (3ci)
Wu (2017)	2014–2016	China	Red deer (*Cervus elaphus*)_	133/410	Serum	ELISA	–	Culture	–
Thiry et al. (2017)	2012	Belgian	Red deer (*Cervus elaphus*)_, Roe deer (*Capreolus capreolus*)	9/424	Serum	ELISA	–	Wild	–
Spancerniene et al. (2018)	2014–2016	Lithuania	Roe deer(*Capreolus capreolus*), Red deer (*Cervus elaphus*), moose (*Alces alces*)	12/71	Serum	RT-qPCR	ORF1/ORF2	Wild	HEV-3 (3i)
Loikkanen et al. (2020)	2008–2009	Finland	Moose (*Alces alces*), White-tailed deer (*Odocoileus virginianus*), Roe deer(*Capreolus capreolus*)	32/424	Serum	ELISA	–	Wild	–
Trogu et al. (2020)	2013–2015	Italy	Red deer (*Cervus elaphus*)	2/254	Serum	ELISA	–	Wild	–
Slukinova et al. (2021)	2018–2019	Russia	Reindeer (*Rangifer tarandus*)	23/191	Serum	ELISA	–	UN	–
Arnaboldi et al. (2021)	2015–2016	Italy	Red deer (*Cervus elaphus*), Roe deer (*Capreolus capreolus*)	0/224	Liver	RT-qPCR	ORF2	Wild	UN
Takahashi et al. (2022)	1997–2020	Japan	Sika deer (*Cervus nippon*)	7/395	Serum and liver	ELISA	–	Wild	–
Moraes et al. (2022)	2018–2020	Portugal	Red deer (*Capreolus capreolus*), Fallow deer (*Dama dama*)	2/130	Faecal	RT-PCR	ORF1	Wild	HEV-3 (3e)
Sacristan et al. (2021)	2010–2018	Norway	Moose (*Alces alces*), Reindeer (*Rangifer tarandus*), Red deer (*Cervus elaphus*), Roe deer (*Capreolus capreolus*)	82/613	Serum	ELISA	–	UN	–
Crotta et al. (2022)	2017–2018	Italy	Red deer (*Cervus elaphus*), Roe deer (*Capreolus capreolus*)	1/323	Serum	ELISA	–	Wild	–
Mendoza et al. (2022)	2008–2021	Japan	Deer (unknown species)	1/2,250	Serum	ELISA	–	UN	–
Barroso et al. (2023)	2005–2006	Spain	Red deer (*Cervus elaphus*), Fallow deer (*Dama dama*)	0/881	Serum	UN	–	UN	–
Cancela et al. (2023)	2020–2022	Uruguay	Spotted deer (*Axis axis*)	6/54	Serum	ELISA	UN	Wild	UN
Sun (2024)	UN	China	Fallow deer (*Dama dama*)	1/99	Faecal	RT-PCR	UN	Wild	UN
Virhuez-Mendoza et al. (2025)	2021–2024	Japan	Sika deer (*Cervus nippon*)	0/583	Serum and faecal	RT-PCR	ORF2	Wild	–
Dudasova et al. (2025)	2015–2020	Slovak	Red deer (*Capreolus capreolus*), Roe deer (*Capreolus capreolus*), Fallow deer (*Dama dama*)	0/99	Liver and muscle	RT-PCR	ORF1/ORF2	Wild	–
Total	–	–	–	1,179/1,2,298	–	–	–		–

UN*: unclear.

Five positive conversions were performed using the data (Supplementary Table S1). Finally, we found that only the “PAS” conversion produced a merged result that most closely approximated a normal distribution (*W* value approaching 1, *p* > 0.05). Therefore, the combined result of the “PAS” conversion was selected for the meta-analysis.

**Figure 2:**
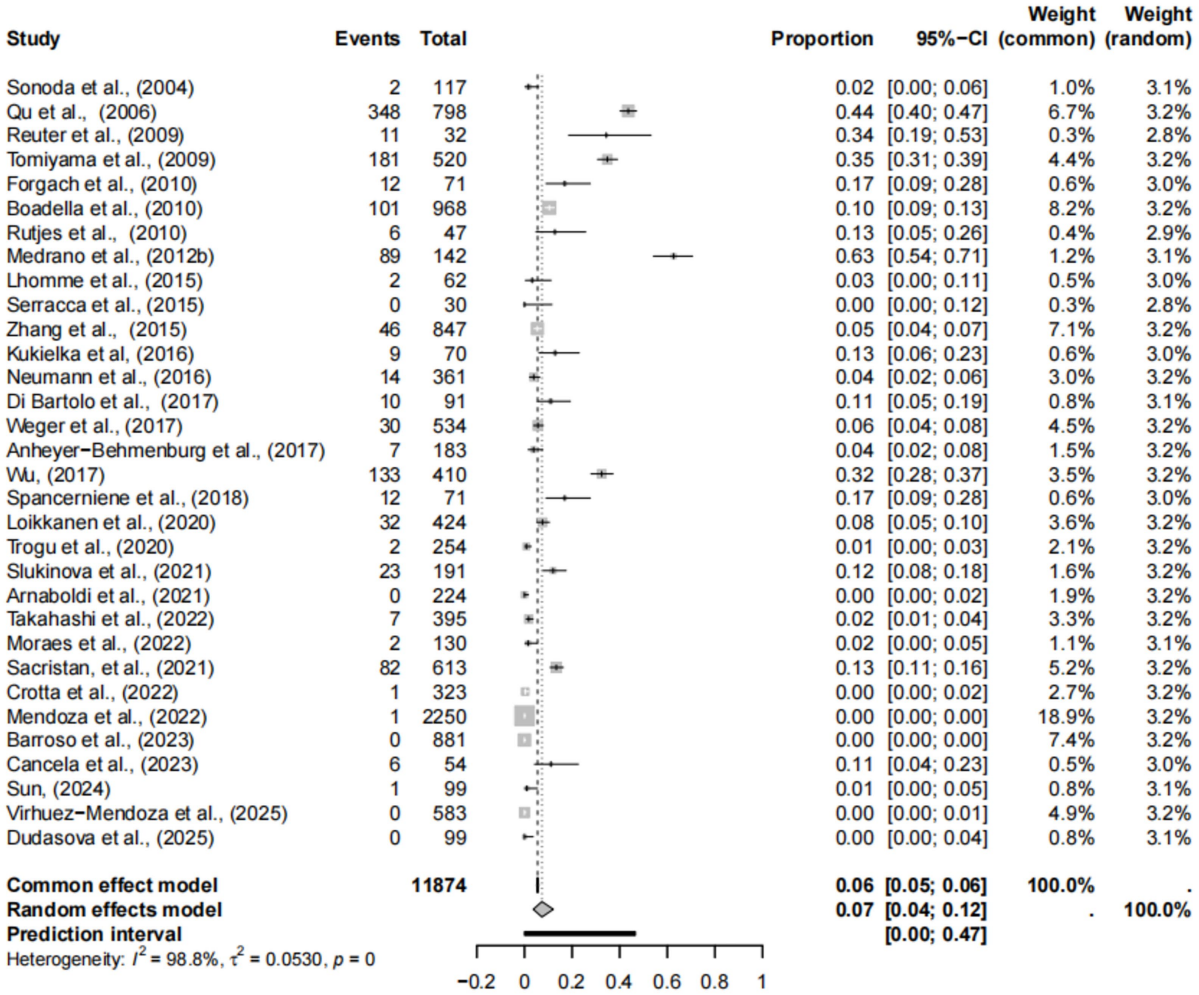
Forest plot of HEV prevalence in deer.

**Figure 3:**
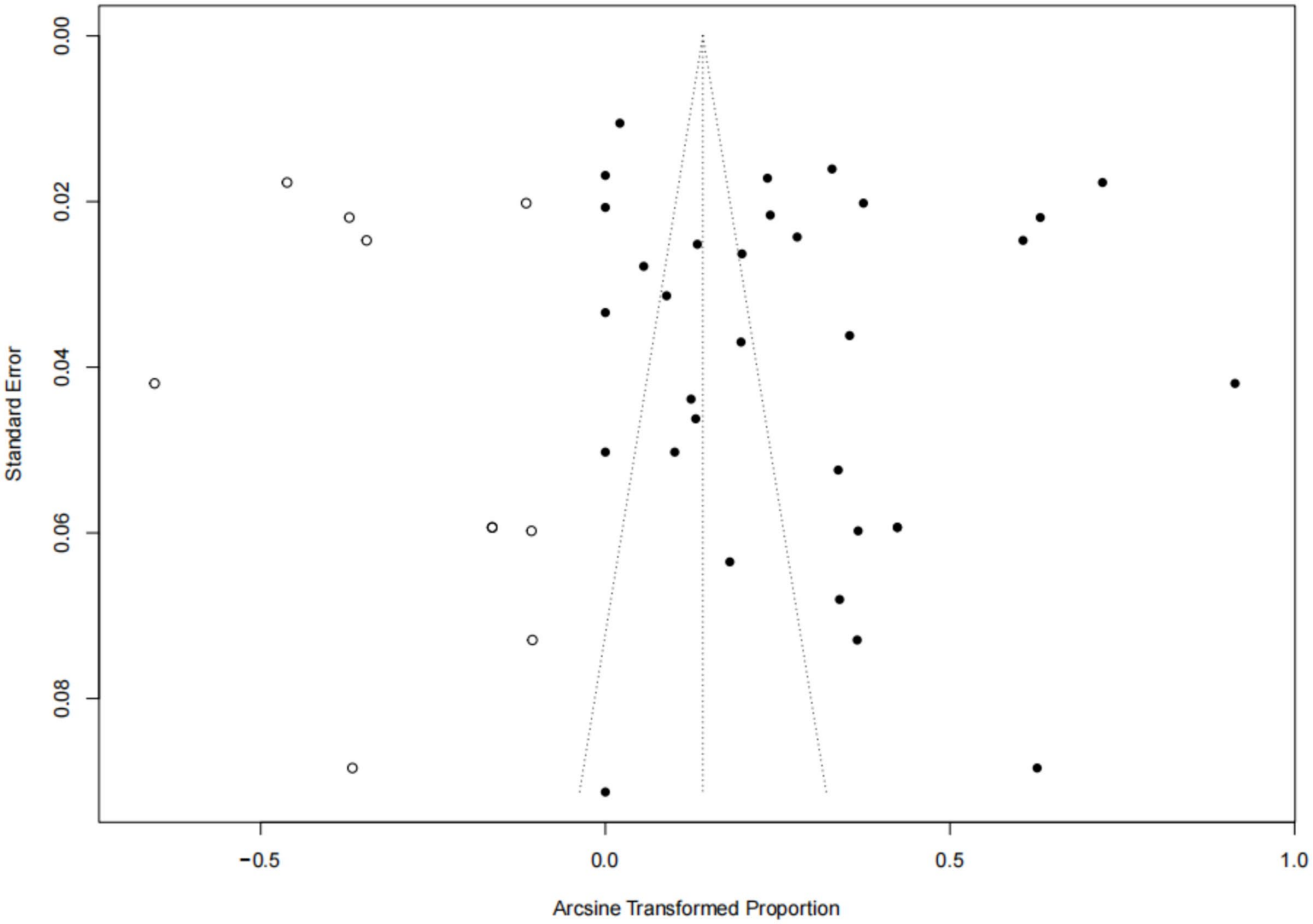
Funnel plot with pseudo 95% confidence interval for publication bias test.

**Figure 4:**
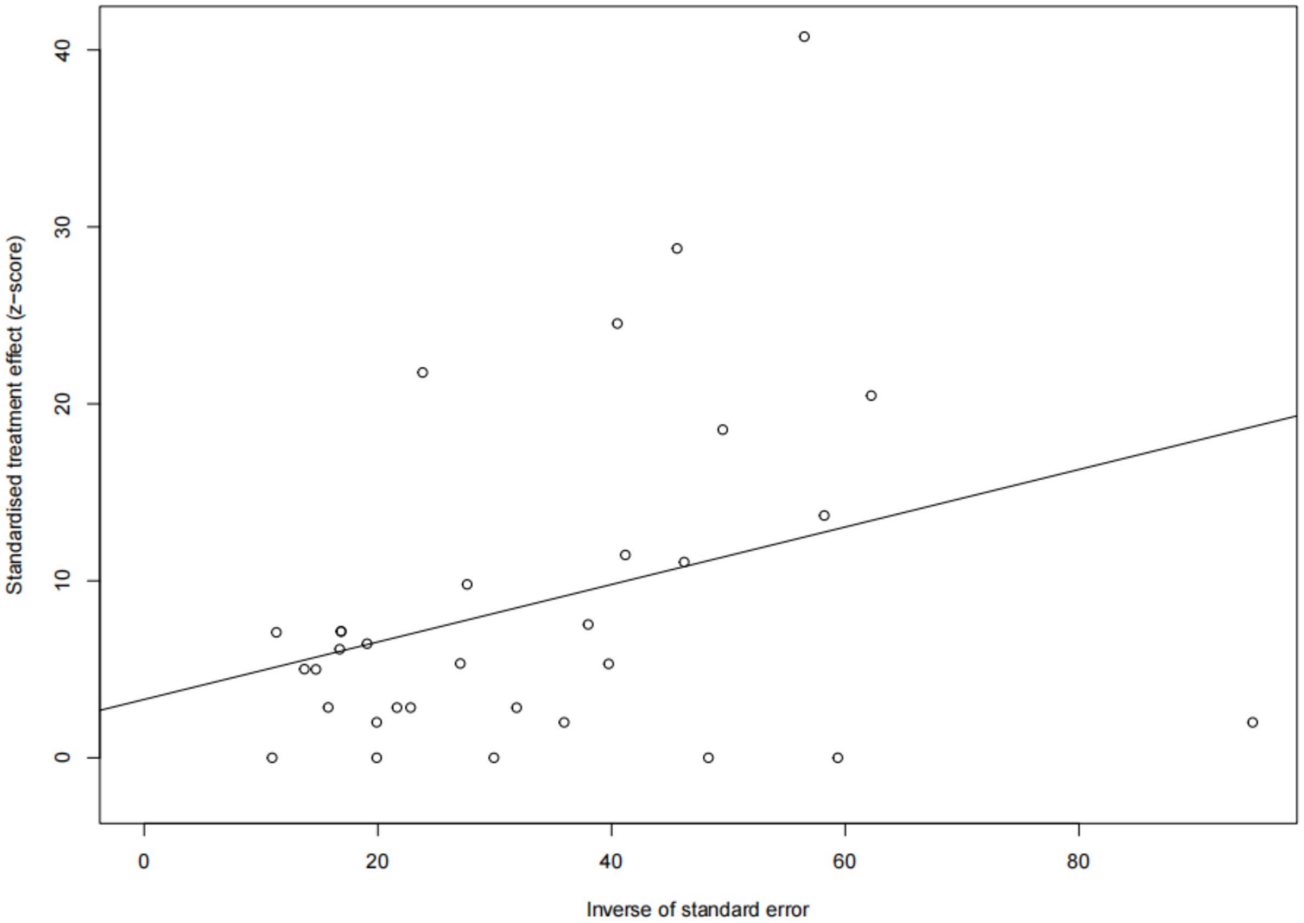
Egger’s test for publication bias.

**Figure 5:**
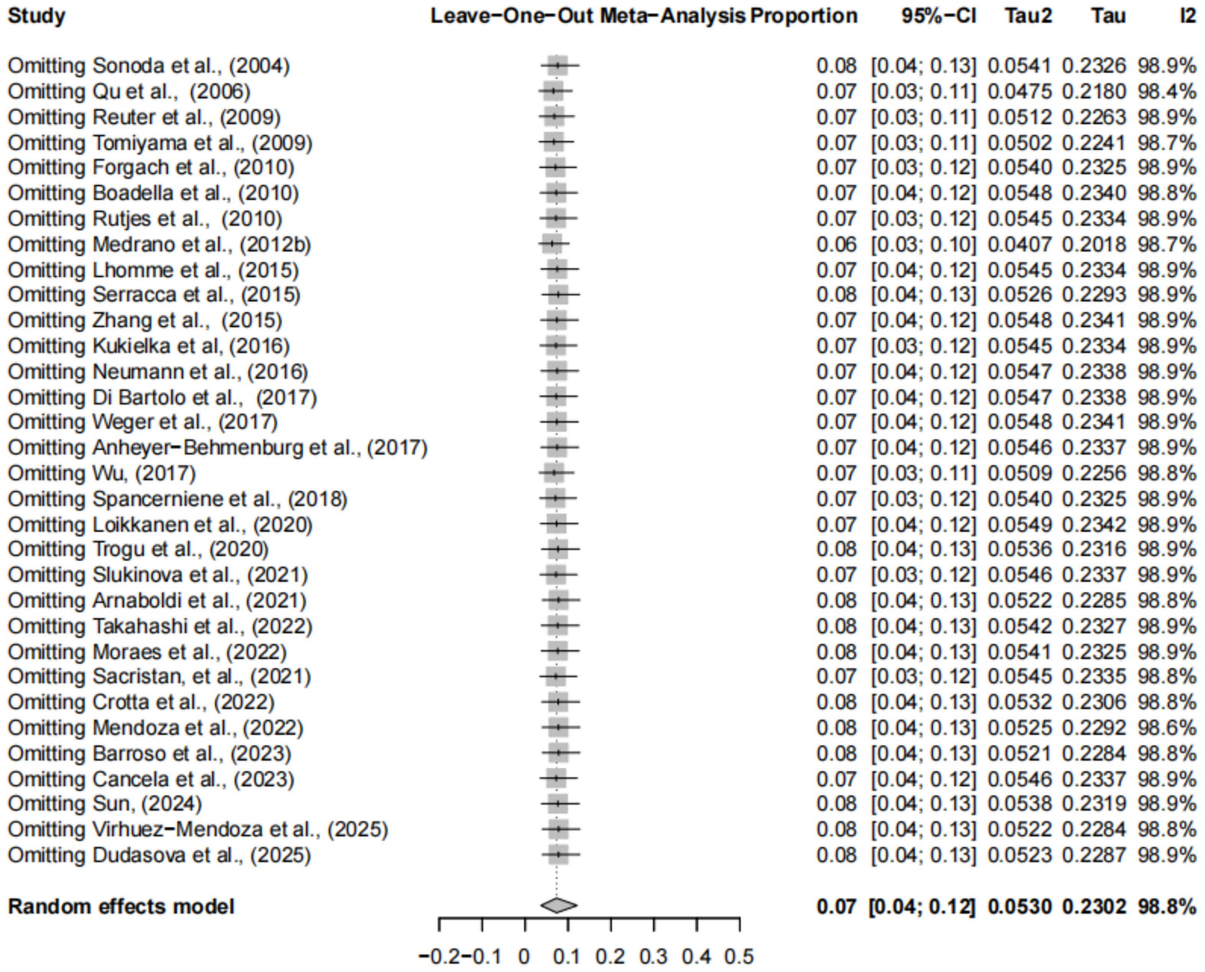
Sensitivity analysis.

Forest plots showed high heterogeneity among the included studies (Figure 2), so the random effects model was adopted. The asymmetry of funnel plot indicates that the results of meta-analysis may be affected by publication bias or small sample bias (Figure 3). Egger test results showed no publication bias (Supplementary Table S2, Figure 4), indicating that the study had no publication bias. The above results indicated that the included study had no publication bias, but there might be a small study effect bias. The results of sensitivity analysis show that the combined data is not affected by any study, thus verifying that the analysis is reasonable and reliable (Figure 5).

### 3.2 Results of subgroup analysis

The combined positive rate of HEV infection in deer was 9.59% (Table 1). The positive rate of white-tailed deer was the highest (19.01, 95% CI: 0.00–62.76) and that of fallow deer was the lowest (0.00, 95% CI: 0.00–0.00). Serological assays (ELISA) detected HEV-specific antibodies with a pooled seroprevalence of 10.48% (95% CI: 4.29–18.92), indicating prior exposure in deer populations. Molecular assays identified viral RNA, with RT-PCR and RT-qPCR yielding pooled detection rates of 8.58% (95% CI: 1.76–19.17) and 5.22% (95% CI: 0.51–13.51), respectively, reflecting evidence of active infection. Subgroup analysis based on feeding practices showed that deer raised under breeding conditions had the highest HEV positivity rate (45.85, 95% CI: 29.21–62.98), whereas wild deer exhibited the lowest prevalence (4.63, 95% CI: 2.02–8.12). The positive rate of female (6.25, 95% CI: 4.38–8.41) was slightly higher than that of male (5.07, 95% CI: 0.79–12.17). The positive rate of adults (23.02, 95% CI: 9.04–41.06) was significantly higher than that of minors (10.11, 95% CI: 5.83–15.41). The continental subgroup showed that the positive rate was highest in North America (29.57, 95% CI: 0.00–89.25) and lowest in Europe (5.55, 95% CI: 2.68–9.27). The positive rate of HEV is higher in developing countries (21.45, 95% CI: 7.12–40.63) than in developed countries (5.01, 95% CI: 2.40–8.43). In the subgroup analysis based on sampling year, studies conducted before 2010 showed a higher overall prevalence (13.17, 95% CI: 6.01–22.45), whereas those conducted in 2010 and beyond exhibited a markedly lower prevalence (4.92, 95% CI: 1.57–9.83) (Table 2). And the country subgroup showed that Mexico had the highest positive rate (62.68, 95% CI: 54.54–70.47) and Slovakia had the lowest positive rate (0.00, 95% CI: 0.00–1.73) (Table 3). By correlation analysis, the heterogeneity of detection methods to other subgroups was 0.00 ~ 46.74% (Tables 2, 3).

**Table 2:** The pooled prevalence of hepatitis E virus in deer.

Risk factor (No. studies)	No. positive/ No. tested	% (95% CI*)	Heterogeneity			Univariate meta-regression		Correlation Analysis
			*χ^2^*	*p*	*I^2^* (%)	p*	Coefficient (95% CI)	*R*^2^-Method
Species						**0.3724** ^**a**^	−0.3777 (−0.6656 to −0.0897)	0.00%
White-tailed deer (3)	108/417	19.01% (0.00–62.76)	163.90	<0.01	98.8%			
Mule deer (1)	5/112	4.46% (1.27–9.22)	0.00	<0.01	NA			
Red deer (16)	283/3,450	4.67% (1.61–8.97)	447.21	<0.01	96.6%			
Sika deer (2)	53/1,242	3.44% (0.77–7.84)	10.44	<0.01	90.4%			
Roe deer (12)	52/823	4.66% (0.48–11.44)	75.15	<0.01	85.4%			
*Dama* (5)	0/1,658	0.0 % (0.00–0.00%)	3.82	0.73	0.0%			
Moose (3)	64/519	12.27% (5.25–21.37)	10.31	<0.01	80.6%			
Reindeer (3)	73/594	11.48% (2.60–25.28)	40.63	<0.01	95.1%			
Spotted Deer (1)	6/54	11.11% (3.88–21.09)	0.00	<0.01	NA			
Serological testing (1)						**0.4610**	−0.0865(−0.3165 to 0.1435)	0.00%
ELISA (17)	1,080/9,107	10.48% (4.29–18.92)	2081.18	0	99.2%			
Molecular biological testing						**0.5262**	0.0522 (0.0051 to 0.1351)	0.00%
RT-PCR (6)	65/888	8.58% (1.76–19.17)	40.84	<0.01	87.8%			
RT-qPCR (5)	27/587	5.22% (0.51–13.51)	44.98	<0.01	91.1%			
Feeding practices						**<0.0001**	−0.5175 (−0.7156 to −0.3194)	46.74%
Wild (23)	344/5,127	4.63% (2.02–8.12)	625.67	<0.01	96.5%			
semi-Wild (1)	101/968	10.43% (8.58–12.44)	0.00	NA	NA			
Farmed (3)	570/1,350	45.85% (29.21–62.98)	41.38	<0.01	95.2%			
Gender						**0.6761**	−0.0242 (−0.1377 to 0.0893)	0.00%
Female (4)	39/600	6.25% (4.38%-8.41)	1.51	0.68	0.0%			
Male (4)	50/890	5.07% (0.79–12.17)	15.16	<0.01	80.2%			
Age						**0.1148**	−0.1725 (−0.3870 to 0.0419)	12.46%
Juvenile (4)	40/404	10.11% (5.83–15.41)	6.74	0.08	55.5%			
Adult (4)	315/1,041	23.02% (9.04–41.06)	109.15	<0.01	97.3%			
Regions						**0.2460**	−0.3222 (−0.6373 to −0.070)	3.28%
North America (2)	119/676	29.57% (0.00–89.25)	202.96	<0.01	99.5%			
Europe (19)	312/4,471	5.55% (2.68–9.27)	268.85	<0.01	93.3%			
Asia (10)	742/6,210	8.65% (1.70–20.00)	1838.53	0	99.5%			
South America (1)	4/56	11.11% (3.88–21.09)	0.00	<0.01	–			
Development level						**0.0030**	0.2509 (0.0853–0.4165)	18.82%
Developing countries (7)	635/2,421	21.45% (7.12–40.63)	571.22	0	98.9%			
Developed countries (25)	544/8,996	5.01% (2.40–8.43)	1052.12	<0.01	97.7%			
Sampling year						**0.0708**	0.2309 (0.1142–0.3476)	10.23%
Before 2010 (17)	851/4,911	0.1317 (6.01–22.45)	115 9.54	<0.01	98.7%			
2010 and beyond (13)	319/3,762	0.0492 (1.57–9.83)	402.78	<0.01	97.0%			

CI*: Confidence interval; p*: There was statistical significance when *p* ≤ 0.05; −*: No Correlation Analysis was performed; ^a^In bold, significant differences.

**Table 3:** Prevalence of HEV in deer in different countries.

Country (No. studies)	No. positive/No. tested	% (95% CI*)	Heterogeneity			Univariate meta-regression		Correlation analysis
			*χ^2^*	*p*	*I^2^*(%)	p*	Coefficient (95% CI)	*R*^2^-Method
Belgium (1)	9/424	2.12 (0.93–3.75)	0.00	NA	NA	**0.2144** ^**a**^	0.7626 (0.1902 to 1.3350)	5.58%
Germany (2)	21/544	3.84 (2.35–5.66)	0.00	1.00	0.0%			
Russia (1)	23/191	12.04 (7.77–17.07)	0.00	NA	NA			
France (1)	2/62	3.23 (0.05–9.48)	0.00	NA	NA			
Finland (1)	32/424	7.55 (5.21–10.27)	0.00	NA	NA			
Holland (1)	6/47	12.77 (4.49–24.05)	0.00	NA	NA			
Canada (1)	30/534	5.62 (3.81–7.74)	0.00	NA	NA			
Lithuania (1)	12/71	16.90 (8.97–26.61)	0.00	NA	NA			
Mexico (1)	89/142	62.68 (54.54–70.47)	0.00	NA	NA			
Norse (1)	82/613	13.38 (10.79–16.19)	0.00	NA	NA			
Portugal (1)	2/130	1.54 (0.02–4.58)	0.00	NA	NA			
Japan (5)	191/3865	3.51 (0.00–16.13)	648.66	<0.01	99.4%			
Spain (3)	110/1919	5.25 (0.00–19.45)	190.75	<0.01	99.0%			
Hungary (2)	23/103	24.14 (9.23–43.00)	3.64	0.056	72.5%			
Italy (5)	13/992	1.04 (0.00–4.85)	27.22	<0.01	85.3%			
China (4)	528/2554	16.73 (1.82–41.92)	464.86	<0.01	99.4%			
Uruguay (1)	0/583	11.11 (3.88–21.09)	0.00	NA	NA			
Slovakia (1)	0/99	0.00 (0.00–1.73)	0.00	NA	NA			

CI *: Confidence interval; NA*: not applicable; *p*-value*: *p* ≤ 0.05 is statistically significant; ^a^In bold, significant differences.

## 4 Discussion

HEV is a major pathogen of acute hepatitis. HEV causes about 20.1 million infections and 70,000 deaths every year, and has become a global public health problem that seriously endangers human health (Rein et al., 2012). HEV infection in animals can cause liver damage in animals, and the meat carries the virus, which can cause HEV infection in humans (Donnelly et al., 2017). Recent studies have also identified raw or unpasteurized milk as a potential vehicle for HEV transmission, as viral RNA has been detected in milk from several animal species and human infections linked to contaminated milk have been reported (Dziedzinska et al., 2020; Hu et al., 2025). The present study conducted a comprehensive global review and meta-analysis on the prevalence of HEV infection in deer populations worldwide. The findings hold immense importance for the prevention and management of HEV in deer populations, as well as for public health.

The included articles were distributed across three regions, with the highest prevalence observed in North America (29.57%), followed by South America (11.11%), Asia(8.65%), and the lowest prevalence recorded in Europe (5.55%). The high prevalence of HEV in North America is consistent with the subgroup analysis conducted on deer species in this study. White-tailed deer, which are primarily distributed in North America, exhibit a higher rate of HEV positivity compared to other deer species. Previous studies have consistently demonstrated that the prevalence of HEV infection in white-tailed deer is three times higher than that observed in red deer. (Medrano et al., 2012). The high infection rate in white-tailed deer is hypothesized to be associated with exposure to wild boars (Weger et al., 2017). The relatively high prevalence observed in white-tailed deer may be influenced by several ecological and biological factors, including habitat characteristics, environmental contamination, and species-specific physiological traits. Evidence from wildlife studies during the COVID-19 pandemic also indicates that white-tailed deer may be particularly susceptible to pathogen exposure under certain environmental conditions, suggesting that similar mechanisms could affect HEV transmission and require further study (Hale et al., 2022).

The results of the country subgroup showed that Mexico had a significantly higher positive rate than other countries (62.68%). This is consistent with the previous epidemic situation in Mexico (Medrano et al., 2012). Since HEV virus was first identified in Latin America, Mexico has become a high-risk area, and the high prevalence in Mexico has become a research hotspot (Realpe-Quintero et al., 2018; Viera-Segura et al., 2023). Evidence from Mexico indicates that human HEV infections are primarily caused by the zoonotic genotypes HEV-2 and HEV-3 (Viera-Segura et al., 2019). Although prevalence in Mexican deer is high, genotype data remain limited because most studies rely on serology rather than sequencing. HEV can be transmitted by water sources, has an interspecific infection, and is susceptible to a variety of animals (Meng et al., 1998; Meng, 2016; Okamoto, 2007). The shedding of viral particles in the stool by subclinically infected individuals may contribute to the persistence of the infection source in Mexico and is also responsible for the high prevalence of HEV infection in Mexican deer (Viera-Segura et al., 2023). China, second only to Mexico (24.75%). China has a long history of breeding deer, and the continued increase in deer numbers in recent years may be a factor in the relatively high prevalence (Li et al., 2013). Other countries such as Hungary (24.14%), Lithuania (16.90), Norse (13.38%), Holland (12.77%), Russia (12.04%) all have prevalence rates above 10%. This indicates that HEV is spreading in deer almost all over the world. The global spread of HEV has been substantiated by previous research, and China has already implemented the use of commercial vaccines to effectively control HEV transmission within its population. Considering these significant outcomes, vaccination against HEV could be a favorable option (Khuroo et al., 2016a; Zhu et al., 2010).

The prevalence of HEV in developed countries (5.01%) is much lower than in developing countries (21.45%). This is in line with prior research, indicating that the prevalence of HEV is significantly influenced by economic and social factors, with a markedly lower positivity rate observed in developed nations compared to developing ones. (Li et al., 2020). The lack of proper sanitation and access to clean drinking water in developing countries is likely to contribute significantly to the prevalence of HEV in human society (Wang and Meng, 2021). As a zoonotic disease, the infection rate of HEV in both farmed and wild deer is also influenced by the overall environmental conditions prevalent in these countries (Pires et al., 2023). Furthermore, it has been demonstrated that transmission of HEV from deer to humans occurs through specific routes (Pires et al., 2023). In addition to prioritizing hygiene and cleanliness in the environment, developing nations should also strengthen their monitoring capabilities for detecting HEV infections in both deer populations and humans (Khuroo et al., 2016b).

The positive rate of samples before 2010 (14.93%) was significantly higher than that after 2010 (4.92%). The World Health Assembly adopted resolution WHA63.18 in 2010, which urged for a comprehensive approach towards the prevention and control of viral hepatitis (HAV, HBV, HCV, HDV, HEV). The adoption of this resolution has raised Member States’ awareness regarding the substantial disease burden associated with viral hepatitis (Diez-Padrisa and Castellanos, 2013). The implementation of the resolution may, to some extent, enhance the efficacy of HEV prevention and control measures and mitigate the rate of HEV transmission.

The results of the sex subgroup showed that the HEV positive infection rate was higher in female deer (6.25%) than in male deer (5.07%). This may be due to differences in the physiological structure and function of females and males, which result in different infection and immune responses to pathogens (Fish, 2008). Females have weaker physical resistance than males, especially after childbirth, resistance decreases and they are more susceptible to HEV infection (Roberts et al., 2001). This also reminds farmers that they should pay attention to the post-natal care of the deer.

The positive rate of farmed deer (45.85%) was significantly higher than that of wild deer (4.63%). Different lifestyles and living environments may lead to different probabilities of contact between deer and pathogens (Colson et al., 2010; Marion et al., 2018). Deer in large-scale breeding environments are more likely to be exposed to potential sources of infection, such as feed and water, etc. (Rogers et al., 2011). The density and contact rate among animals increase, and deer feces cannot be cleaned up in time, resulting in a higher HEV infection rate in captive deer (Fong, 2017). It is also easy to cause HEV transmission and infection in animals, so it is recommended to reduce breeding density and improve animal welfare.

Differences in HEV prevalence across diagnostic methods should be interpreted in light of their biological targets. The results of the subgroup of detection methods showed that enzyme-linked immunosorbent assay (ELISA) had the highest infection rate (10.48%), followed by RT-PCR (8.58%). Among these detection methods, ELISA is suitable for large-scale detection of infections that have already produced an immune response (Liu et al., 2015), but sometimes the sensitivity of reagent anti-HEV IgM detection is low, and some studies have reported that existing diagnostic reagents lack the reactivity of serum anti-HEV against some infected genotypes of patients (Wang and Zhuang, 2004). In this subgroup, RT-PCR detection is the most direct evidence of acute HEV infection, which can be detected using acute serum or feces, contaminated water, sewage, etc. (Wen, 2009), but the tolerance of HEV nucleic acid to the environment is poor, and the amount of virus in both serum and feces decreases rapidly after the onset of HEV. Therefore, the requirements for sample collection and preservation are very high (Wen, 2009). RT-qCR detects low concentrations of target molecules and is capable of accurate quantitative analysis (Shaoquan, 1990). At the same time, the amplification process can be monitored in real time during the reaction process to reduce experimental errors (Bustin, 2002). However, there is currently no universally accepted gold standard method for detecting HEV, thus necessitating the development and evaluation of a precise detection method as well as the establishment of an internationally recognized HEV serological reference standard in order to accurately determine its prevalence (Pisano et al., 2022).

These results, to some extent, reflect the prevalence of HEV in deer around the world. In this study, there were ten 2-point articles and 17 3-point articles, with the main loss of marks on “whether there is sex,” followed by the failure to mention the growth stage of the host and the sampling method. Therefore, it is suggested that researchers should collect more samples when conducting epidemiological investigations, and record in detail the information such as gender, age and breed. Sampling time and location are also important information, which can provide basic time and location information, explore more influencing factors, and provide more data for the prevention and treatment of HEV infection in deer.

In summary, understanding the risk factors associated with HEV infection in deer is crucial for effective prevention and control of HEV transmission. This study aimed to identify key risk factors for deer HEV, such as national development level and regional distribution. Additionally, the prevalence rate was found to be closely linked to the survival mode, sex, and age of deer. However, due to variations in detection methods leading to heterogeneity, there is an urgent need for a standardized gold standard method for HEV detection. This will enable a better understanding of the burden posed by this emerging and poorly understood pathogen, as well as identifying modifiable risk factors that can be targeted for preventing further infections.

## Data Availability

The original contributions presented in the study are included in the article/Supplementary material, further inquiries can be directed to the corresponding authors.
