# Supplementary material for: Meta-analysis of global prevalence of hepatitis E virus infection in deer
Source: Front Microbiol. 2025 Dec 19;16:1741279. doi: 10.3389/fmicb.2025.1741279 (PMC12757369; doi:10.3389/fmicb.2025.1741279)
Supplement: Supplementary file 1 [file Data_Sheet_1.docx]

**Table S1**

Normal distribution test and different conversion of normal prevalence.

| **Conversion form** | **W** | **P** |
| --- | --- | --- |
| PRAW | 0.7497 | 5.243e - 06 |
| PLN | NAN | NA |
| PLOGIT | NAN | NA |
| PAS | 0.91379 | 0.01415 |
| PFT | 0.91794 | 0.01829 |

"PRAW": raw exchange rate; PLN: log conversion. "PLOGIT": logit transformation;

"PAS": arcsine transformation; "PFT": double arcsine transformation;

**Table S2**

Egger’s for publication bias.

| **slope** | **bias** | **se. bias** | **t** | **df** | ***p-value*** |
| --- | --- | --- | --- | --- | --- |
| 0.0936 | 4.2066 | 3.7188 | 1.13 | 26 | 0.2683 |
